# Local dynamics of proteins and DNA evaluated from crystallographic *B* factors

**DOI:** 10.1107/S1399004714014631

**Published:** 2014-08-29

**Authors:** Bohdan Schneider, Jean-Christophe Gelly, Alexandre G. de Brevern, Jiří Černý

**Affiliations:** a Institute of Biotechnology AS CR, Videnska 1083, 142 20 Prague, Czech Republic; b INSERM, U1134, DSIMB, 75739 Paris, France; c Université Paris Diderot, Sorbonne Paris Cité, UMR_S 1134, 75739 Paris, France; d Institut National de la Transfusion Sanguine (INTS), 75739 Paris, France; e Laboratoire d’Excellence GR-Ex, 75739 Paris, France

**Keywords:** *B* factors, local dynamics

## Abstract

The dynamics of protein and nucleic acid structures is as important as their average static picture. The local molecular dynamics concealed in diffraction images is expressed as so-called *B* factors. To find out how the crystal-derived *B* factors represent the dynamic behaviour of atoms and residues of proteins and DNA in their complexes, the distributions of scaled *B* factors from a carefully curated data set of over 700 protein–DNA crystal structures were analyzed [Schneider *et al.* (2014 ▶), *Nucleic Acids Res.* **42**, 3381–3394]. Amino acids and nucleotides were categorized based on their molecular neighbourhood as solvent-accessible, solvent-inaccessible (*i.e.* forming the protein core) or lying at protein–protein or protein–DNA interfaces; the backbone and side-chain atoms were analyzed separately. The *B* factors of two types of crystal-ordered water molecules were also analyzed. The analysis confirmed several expected features of protein and DNA dynamics, but also revealed surprising facts. Solvent-accessible amino acids have *B* factors that are larger than those of residues at the biomolecular interfaces, and core-forming amino acids are the most restricted in their movement. A unique feature of the latter group is that their side-chain and backbone atoms are restricted in their movement to the same extent; in all other amino-acid groups the side chains are more floppy than the backbone. The low values of the *B* factors of water molecules bridging proteins with DNA and the very large fluctuations of DNA phosphates are surprising. The features discriminating different types of residues are less pronounced in structures with lower crystallographic resolution. Some of the observed trends are likely to be the consequence of improper refinement protocols that may need to be rectified.

## Abstract 2

Distributions of scaled *B* factors from 704 protein–DNA complexes reflect primarily the neighbourhood of amino-acid and nucleotide residues: their flexibility grows from the protein core to protein–protein and protein–DNA interfaces, to solvent-exposed residues. Some of the findings clearly observed at higher resolution structures can no longer be observed for structures at low resolution indicating problems in refinement protocols.

## 1. Introduction   

Crystallographic *B* factors (Trueblood *et al.*, 1996 ▶; Rupp, 2009 ▶) represent the uncertainty in atom positions in the refined model that results from the superposition of atomic vibrations and crystallographic disorder. Accurate determination of crystal structures requires the proper treatment of *B* factors during the refinement process (Tronrud, 1996 ▶; Merritt, 2012 ▶), but the validation criteria for *B*-factor values are much less sophisticated than those for the validation of atomic coordinates, so that *B*-factor values might also reflect errors in diffraction data and their incorrect treatment during the refinement process.

The definition of *B* factors implies that they are a measure of local atomic movements. To determine whether and to what extent they can be used as a realistic gauge of the local molecular dynamics at the subnanometre scale, we decided to analyze their distributions in over 700 crystal structures of protein–DNA complexes selected from a larger well curated data set previously used for the analysis of the structural properties of protein–DNA complexes (Schneider *et al.*, 2014 ▶). We were primarily interested in comparing the flexibility of residues of these key biomolecules in different types of molecular environment: exposed to solvent or surrounded by residues from the same or neighbouring biomolecules; we also wanted to juxtapose the flexibility of biopolymer residues and supposedly the least restricted molecules, waters. A simple statistical analysis of *B*-factor distributions in these structures confirmed several expected features of the local dynamics of proteins and DNA, but also revealed some surprising observations. To the best of our knowledge, a comprehensive bioinformatics analysis of the behaviour of *B* factors based on a large and structurally diverse ensemble of hundreds of structures at a wide range of crystallographic resolutions has not been performed as yet, and we therefore believe that the present bioinformatics study offers some generally valid conclusions about the behaviour of *B* factors and its relationship to biomolecular dynamics.

## 2. Materials and methods   

### 2.1. Selection of protein–DNA structures   

We analyzed a data set of protein–DNA complexes retrieved from the Nucleic Acid Database (Berman, Westbrook *et al.*, 2002 ▶) and the Protein Data Bank (Berman, Battistuz *et al.*, 2002 ▶); X-ray structures containing protein, DNA longer than five nucleotides and no RNA were selected. The data set was curated as described in detail previously (Schneider *et al.*, 2014 ▶). The original nonredundant data set contained 1018 complexes with crystallographic resolution better than 3.3 Å. For the purpose of our *B*-factor analysis, we reduced this limit to 3.0 Å in order to remove structures with the lowest resolution; the number of complexes was 949. To exclude a possible role of the data-collection temperature in *B*-factor distributions, we excluded 147 structures with a data-collection temperature that was unreported or above 180 K. Finally, so as not to confuse proper *B* factors and so-called residual *B* factors resulting from refinement algorithms using the TLS (translation/libration/screw) concept (Howlin *et al.*, 1993 ▶), we also excluded 93 structures of protein–DNA complexes refined using TLS protocols; the list of PDB files with partial *B*-factor values was downloaded from the RCSB site (ftp://ftp.wwpdb.org/pub/pdb/doc/revision_logs/). All in all, we analyzed 709 protein–DNA complexes: 165 with resolution 1.9 Å and better (labelled R1), 357 structures with resolution between 1.9 and 2.5 Å (labelled R2) and 187 structures with resolution between 2.5 and 3.0 Å (labelled R3).

### 2.2. Classification of residues based on their neighbourhood   

Amino-acid and nucleotide residues were classified according to their molecular and crystal neighbourhood. We identified six types of protein residues and two types of DNA residues. Amino acids with less than 5% of their surface exposed to solvent were classified as ‘buried aa’ and those with more than 35% of their surface exposed to solvent were classified as ‘exposed aa’; partially accessible amino acids with a solvent exposure of between 5 and 35% were labelled as ‘partially buried aa’, but are not further discussed here. Three remaining classes of amino acids were assigned based on their interaction with other biopolymers. Amino acids interacting with amino acids from a different protein molecule (from a different protein chain) inside the asymmetric unit were classified as ‘protein–protein aa’; amino acid–amino acid contacts across the symmetry (contacts outside the asymmetric unit) were classified as ‘protein–symprotein aa’. Amino acids interacting directly with nucleotides were classified as ‘protein–DNA aa’; amino-acid residues bound to a nucleotide *via* a water bridge were not included in this group. The DNA molecule, with no solvent-hidden interior, is topologically simpler and we therefore generated only two classes of nucleotides: those in direct contact with protein, which were labelled ‘DNA–protein nt’, and those interacting solely with other nucleotides *via* base pairing and otherwise exposed to solvent, which were labelled ‘exposed nt’. In all classes of amino-acid and nucleotide residues, we analyzed the backbone and side-chain atoms separately. In DNA, the phosphate and deoxyribose atoms were considered to be the backbone and the atoms of the nitrogenous bases were considered to be the side chains. It should be noted that two classes of amino-acid residues, protein–protein aa and buried aa, are not completely exclusive as some amino acids at the interface with another protein molecule may be inaccessible to solvent. All other classes are exclusive.

In addition, we analyzed two classes of ordered water molecules. The first was those that are bound to no more than one polymer atom and therefore do not bridge two polymer residues; they are called ‘surface w’. The other type of analyzed water molecules is formed by water molecules bridging an amino-acid residue with a nucleotide residue and are called ‘bridge w’. Water molecules bridging two protein chains were not explicitly analyzed; we also did not analyze biopolymer residues bridged by a water molecule.

The solvent accessibility was calculated by the *VMD* (*Visual Molecular Dynamics*) program (Humphrey *et al.*, 1996 ▶) considering the geometry of the whole complex including symmetry-related molecules. Two residues were considered to be in contact if their non-H atoms were closer than 3.40 Å. Contacts were calculated by in-house scripts employing the *VMD* program (Humphrey *et al.*, 1996 ▶). Both direct and water-mediated contacts were determined considering the crystallographic symmetry. Symmetry-related atoms were generated using a modified version of the *GENSYM* program from the *CCP*4 suite (Collaborative Computational Project, Number 4, 1994 ▶; Winn *et al.*, 2011 ▶); the symmetry operators were taken from the PDB coordinate files. The symmetry-related atoms were generated up to 10 Å from atoms in the asymmetric unit using the *VMD* program.

### 2.3. Scaling of *B* factors   

*B* factors were extracted from the analyzed structures, but direct comparison of their values was not possible because they were scaled differently in different structures owing to the use of different refinement strategies. We employed the two most frequently used scaling procedures: (i) unity-based scaling and (ii) *z*-score normalization. Unity-based normalization scales variables, here *B* factors, between one common minimum value and one common maximum value. For each structure in the data set, we set the lowest *B* factor to 1.0 and the highest *B* factor to 100.0; intermediate *B*-factor values were scaled linearly between these values. The scaled *B* factor *B*_*x*-scaled_ for atom *x* in structure *i* was calculated according to the formula
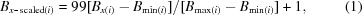
where *B*_*x*(*i*)_ is the *B* factor of atom *x* in the structure (*i*) and *B*_min(*i*)_ and *B*_max(*i*)_ are the *B* factors with the minimal and maximal values in the structure (*i*), respectively. The scaled atomic *B* factors *B*_*x*-scaled(*i*)_ were then separately averaged for the backbone and side-chain atoms of each residue and the values of these scaled residue-averaged *B* factors were used to compare the fluctuations of amino acids, nucleotides and water molecules.

*B* factors in individual structures were also normalized by the frequently used ‘*z*-score normalization’,
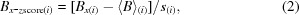
where 〈*B*〉_(*i*)_ is the arithmetical average of the *B* factors in structure (*i*) and *s*_(*i*)_ is the corresponding estimated standard deviation. As in the unity-based scaling, individually *z*-score-scaled *B* factors *B*_*x*-*z*score(*i*)_ were separately averaged for the backbone and side-chain atoms of each residue and the values of these scaled residue-averaged *B* factors were used to compare the fluctuations of amino acids, nucleotides and water molecules.

No other manipulations of the *B*-factor values were carried out. Specifically, we did not remove extremely large values or outliers, no matter how they were defined, because we wanted to describe the distributions as they have been reported and to eventually emphasize the differences between different residues, and not determine the optimal or ‘correct’ parameters for flexibility of amino acids as did the authors of previous work (Smith *et al.*, 2003 ▶).

## 3. Results and discussion   

### 3.1. Populations of the analyzed types of residues   

**Figure 1:**
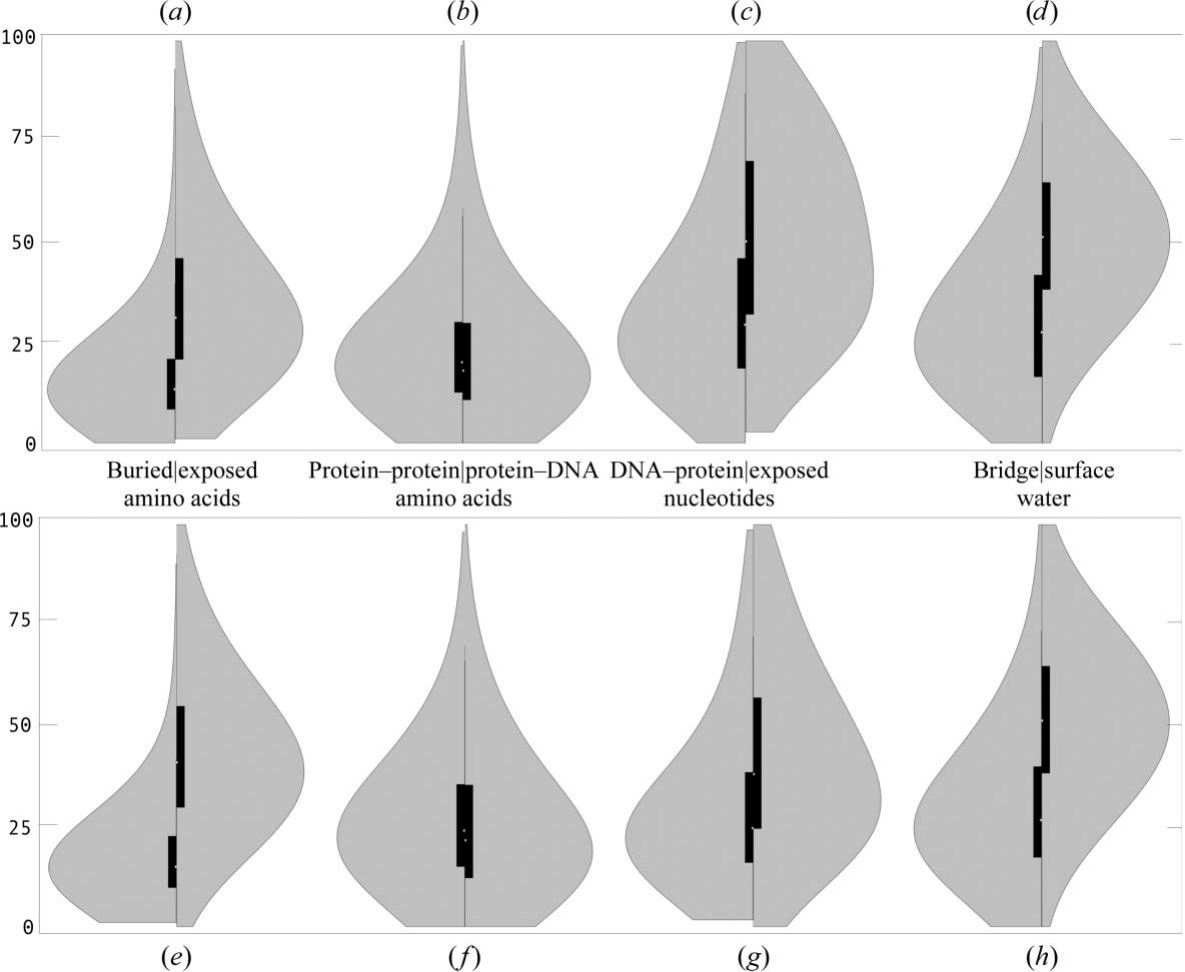
Distributions of scaled *B* factors in the group of high-resolution protein–DNA complexes (165 structures; bin R1). (*a*)–(*c*) show distributions for atoms of the protein and DNA backbone (BB) and (*e*)–(*g*) show those for the amino-acid side chains and nucleotide nitrogenous bases (SC). (*d*) compares surface waters with waters bridging amino acids and DNA phosphates and (*h*) compares surface waters with waters bridging amino acids and DNA bases. Smoothed histograms are plotted in grey, black boxes show the second and third quartiles and the white spot indicates the median. (*a*, *e*) buried aa *versus* exposed aa, (*b*, *f*) protein–protein aa *versus* protein–DNA aa, (*c*, *g*) DNA–protein nt *versus* exposed nt, (*d*, *h*) bridge w *versus* surface w. The residue classes for which the histograms were plotted are also indicated between the two panels. Analogous distributions for all the three resolution bins R1, R2 and R3 are shown in Supplementary Figs. S1(*a*)–S1(*f*).

**Figure 2:**
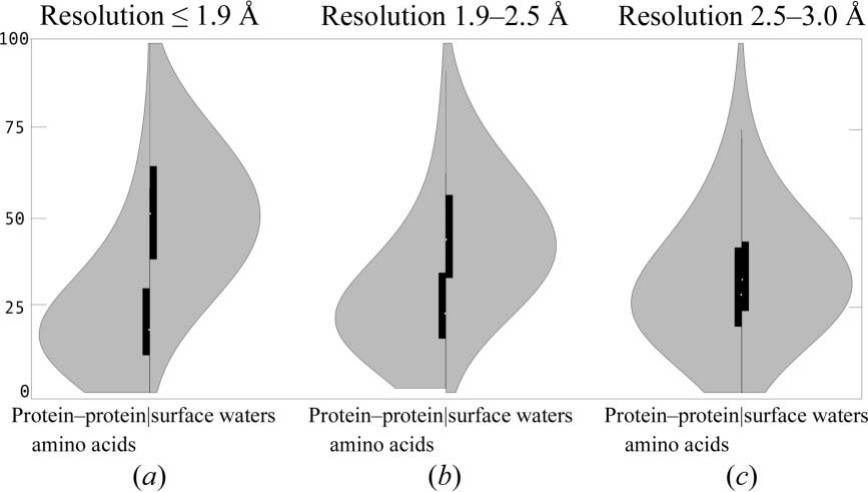
Comparison of normalized *B*-factor distributions in the three resolution bins for the backbone atoms of amino-acid residues in contact with another protein (the protein–protein aa group of residues) and the first-shell water molecules (the surface w group).

Table 1 ▶ shows the populations of the classified groups of amino-acid, nucleotide and water residues as found in 709 crystal structures of protein–DNA complexes in the three resolution bins. The numbers of all classes of residues are sufficiently large in all resolution bins to carry out statistical analysis. We plotted the distributions of *B*-factor values as smoothed histograms scaled by the unity-based algorithm (equation 1; Figs. 1 ▶ and 2 ▶ and Supplementary Fig. S1^1^) as well as by the *z*-score algorithm (equation 2; Supplementary Fig. S2) and calculated their basic statistics (Supplementary Table S1). In Fig. 1 ▶, we compare the dynamic behaviour of eight residue classes in the highest resolution bin R1; in Fig. 2 ▶, we juxtapose the dynamics of two groups of residues, protein-protein aa and surface w, and show how differently they behave in the resolution bins R1, R2 and R3. The histograms were plotted ‘back-to-back’ to stress the (dis)similarity of two directly compared distributions.

### 3.2. The dynamics of various types of residues in the highest resolution bin   

Fig. 1 ▶ shows distributions of the unity-based scaled *B* factors for the structures in the high-resolution bin R1: the backbone (main-chain) atoms (BB) are shown in Figs. 1 ▶(*a*)–1 ▶(*d*) and the side-chain atoms (SC) are shown in Figs. 1 ▶(*e*)–1 ▶(*h*). The histograms demonstrate that each residue class presents a distinct pattern of dynamic behaviour. A large contrast was observed between buried and solvent-accessible amino acids (Figs. 1 ▶ *a* and 1 ▶ *e*; buried aa *versus* exposed aa) and notably also between water molecules forming the protein–DNA bridges and the first-shell waters of biopolymers (Fig. 1 ▶ *d*; bridge w *versus* surface w).

#### 3.2.1. *B*-factor distributions of the backbone atoms   

The atoms of the buried amino acids, buried aa, behave differently to any other group of residues. Their displacements are distinct in terms of both the overall distribution shape and its numerical characteristics (Table 1 ▶; further data are in given in Supplementary Table S1): the *B* factors of both BB and SC atoms of buried aa are concentrated at low values and possess a very thin high-value tail; the median value for BB atoms in buried aa is 14. Amino acids interacting with another protein chain or with DNA (Figs. 1 ▶ *b* and 1 ▶ *f*; protein–protein aa and protein–DNA aa) have mutually similar distributions. The atoms of these residues are much more flexible than the atoms from buried aa, and their whole populations are shifted to higher *B* values, with a correspondingly large median value of around 20. Solvent-exposed amino acids at the protein surface, exposed aa, are still more flexible, with a *B*-factor median twice as large as that for buried aa. Exposed aa can be quite rigid but also extremely flexible, as is shown by their almost symmetrical *B*-factor distribution with a large variance. Overall, the backbone atoms of amino acids are most restricted in the protein core, more flexible at the interfaces with other biopolymers and much more flexible when exposed to solvent.

Atoms of the DNA phosphodiester backbone have high flexibility at the interface with proteins but especially when fully exposed to solvent. DNA backbones interacting with proteins are much more flexible than their amino-acid counterparts; the respective median values are 31 and 19, but the character of both distributions is similar. The DNA backbone atoms exposed to solvent, exposed nt, have a flat distribution with many extremely high *B* values and a median of 51. Unexpectedly, this is the same value as the *B*-factor median of the least constrained atoms in our data set: the first shell waters, surface w. Perhaps more surprising is the observation that the atomic displacements of water molecules bridging DNA and proteins, bridge w, are similar to those of DNA–protein nt and exposed aa: all three groups of atoms have a median value of around 30.

#### 3.2.2. *B*-factor distributions of the side-chain atoms   

An overall comparison between the distributions of the backbone and side-chain atoms (Figs. 1 ▶ *a*–1 ▶ *d* *versus* Figs. 1 ▶ *e*–1 ▶ *h*) shows a qualitative difference between the behaviour of proteins and DNA: while the amino-acid side chains are generally slightly more floppy than the backbones, the phosphodiester backbone is much more flexible than the atoms of the nitrogenous bases.

Of all of the *B*-factor distributions, those for the buried amino acids are unique: not only are they the tightest, but also their side-chain and backbone atoms are equally rigid so that the known tight packing of the protein core (Richards, 1974 ▶; Chothia, 1975 ▶) limits the flexibility of potentially floppy side chains to a similar extent as the flexibility of a conformationally more restricted backbone. Restrictions on the atomic displacements in the solvent-inaccessible core are perhaps more obvious when compared with the displacements of amino acids involved in protein–protein and protein–DNA inter­actions. Most of these amino acids mediate protein–protein or protein–DNA recognition and are therefore quite specific. Their flexibility is much higher than that of amino acids in the protein core. *B*-factor distributions can be used here as a proxy for the ‘density of interaction’, which is highest in the protein core, lower for the intermolecular recognition region and lowest at the solvent boundary. It correlates well with the results of Halle (2002 ▶), who tested the hypothesis that *B* factors are inversely proportional to the local packing density, which is basically the number of noncovalent neighbour atoms within a volume of approximately 1.5 nm^3^. His analysis of 38 proteins resolved at high crystallographic resolution led to the conclusion that the *B*-factor profile is essentially determined by spatial variations in local packing density, and Halle predicts an approximately directly inverse relationship between *B* factors and the packing density and concludes that *B* factors provide little independent information beyond that contained in the mean atomic coordinates.

It is worthwhile noting that the backbone and side-chain distributions of all types of amino-acid residues have a similar character. This is especially the case for amino acids buried in the protein interior and involved in interaction with DNA; however, even amino acids exposed to solvent have main-chain and side-chain atoms of comparable flexibility (median values of 32 and 41, respectively). The side chains of amino acids interacting with DNA are of particular interest here; the extended side chains of arginine and lysine form about 50% of these contacts, yet the *B*-factor distributions of the main-chain and side-chain atoms are almost the same. A deeper residue-type analysis may reveal differences in flexibility between different amino acids, especially on the protein surface, but in-depth sequence-dependent analysis of *B*-factor distributions is beyond the scope of this study.

The nitrogenous bases of DNA are much less flexible than the DNA phosphodiester backbone. Bases from the group of nucleotides exposed to solvent, exposed nt, have a *B*-factor median of 38, similar to the side chains of solvent-exposed amino acids. The flexibility of the bases is, however, quite high considering the fact that virtually all bases form pairs with bases from the other DNA strand of the duplex: very few DNA strands in our sample of protein–DNA complexes are single-stranded, while a few are tetraplexes. Bases from the exposed nt group are actually more flexible than water molecules forming DNA–protein bridges, bridge w. These bridge water molecules have a flexibility comparable to that of DNA bases from nucleotides in direct contact with protein, DNA–protein nt (median values are given in Supplementary Table S1). This observation is perhaps surprising considering the fact that it was drawn from the structures in the high-resolution bin R1, which have the highest ratio between the experimental data (structure factors) and refined parameters (coordinates and *B* factors) and thus provide the best estimate of the inherent dynamic properties of molecules. The relatively low flexibility of the bridge waters, comparable to that of DNA bases, stresses their importance in protein–DNA recognition and is in agreement with our earlier observation that protein–DNA interfaces formed by direct amino acid–nucleotide contacts or *via* water bridges have similar structural features (Schneider *et al.*, 2014 ▶).

We analyzed the *B*-factor distributions of nucleotides bound to proteins in greater detail and calculated *B* factors separately for those interacting with proteins by the phosphate and base atoms, respectively. However, these distributions are virtually identical, so that the nucleotide flexibility is restricted to a similar extent regardless of whether it binds to a protein residue by its phosphate or base. Stiffening of nucleotides upon protein binding can be generalized a step further: it is almost the same whether protein and DNA interact by direct polar contacts or *via* a water bridge. This finding is less obvious and further supports the importance of water bridges for recognition.

### 3.3. The effect of crystallographic resolution on the distribution of normalized *B* factors   

All of the differences between various types of residues are most pronounced for structures in the high-resolution bin R1; lowering the resolution removes the differences between the different types of residues. The distributions of the scaled *B* factors in the three resolution bins are compared in Fig. 2 ▶ between two residue classes, protein–protein aa and surface w; all residue classes in the three resolution bins are shown in Supplementary Fig. S1. Comparison of Figs. 2 ▶(*a*) and 2 ▶(*c*) shows a large contrast: while the distributions of both of the types of residues are quite different for structures in the high-resolution bin R1, they become almost indistinct in the lowest resolution bin R3. The loss of distinction between different types of residues at lower resolutions is also obvious when we look at the median values in Table 1 ▶ (further data are given in Supplementary Table S1). For instance, while the difference between buried aa and bridge w is twofold in the R1 bin, the values are the same in the R3 bin; in addition, the highest medians were observed in the low-resolution bin for the side chains of exposed aa and exposed nt, not for surface w.

One feature distinguishes the *B*-factor distributions of the polymer residues and waters: while the former values shift to higher *B* values at lower resolution, the latter remain about the same or even decrease: the median of surface w is 51 in the R1 bin and 33 in the R3 bin. The lower displacements of water molecules observed at lower resolutions are contrary to intuitive expectations. A simple explanation might perhaps lie in the fact that only a few of the best-ordered water molecules are refined at low resolutions and naturally these have low *B* factors. However, this explanation is not fully satisfactory because one would still expect that at least the protein backbone atoms connected in sterically restricted polymer chains would fluctuate less than the less restricted water molecules.

The behaviour of *B* factors in the protein core poses perhaps an even more pressing question. They have distinct distributions in high-resolution structures but become indistinguishable from residues at the interface of other proteins or DNA: is the unique behaviour of the protein interior really lost at lower resolutions or is the vanished unique behaviour a consequence of improper refinement? We believe that the above-described blurring of the differences between different types of residues on lowering the resolution (distributions are shown in Supplementary Figure S1 and statistics are given in Supplementary Table S1) hardly reflects the true behaviour of solvated protein–DNA complexes and can be attributed to unsuitable refinement protocols. Whereas diffraction data obtained at relatively high resolutions of better than 1.9 Å lead to *B*-factor distributions with apparently reasonable properties, the fewer data obtained at lower resolutions do not contain enough information to impose sufficient constraints to determine independent *B* values for all atoms, and the *B*-factor values of different types of residues become similar. That *B* factors are not completely independent and physically meaningful quantities has been indicated by Weiss (2007 ▶): up to half of the total *B*-factor variation in macromolecular structures may be successfully predicted based solely on the atomic coordinates and just three additional parameters per structure; the results of Halle (2002 ▶) indicating a limited information content of *B* factors have already been discussed.

### 3.4. *B*-factor distributions for complexed and uncomplexed residues   

The standardized *B*-factor distributions reflect important general properties of the analyzed complexes. Regardless of resolution, the mutual interaction between protein and DNA molecules stiffens protein and DNA atoms to a similar extent. ‘Cooling off’ of the interacting residues upon complexation reduces the median values of the standardized *B* factors by about 50–75% for both the backbone and side chains. Such a significant lowering of atomic fluctuations has its entropic cost and inevitably impacts protein–DNA or protein–protein affinity by lowering the free-energy gain of the interaction. While the differences between the *B*-factor distributions of different residues becomes blurred at lower resolutions, the differences between complexed and uncomplexed residues remain significant even in the lowest resolution bin. By extrapolation, one might assume that this effect remains important even for the interaction between partners in solution.

### 3.5. Comparison of *B* factors scaled using the unity-based and *z*-score formulas   

All of the analyses presented so far were based on distributions obtained by unity-based scaling (1). The general characteristics of the distributions remained the same when we inspected distributions calculated by *z*-score normalization according to (2). Because the alternative normalization did not reveal any new features of the behaviour of the *B* factors, the distributions are presented in Supplementary Fig. S2: Supplementary Fig. S2(*a*) shows the distributions of the backbone atoms and Supplementary Fig. S2(*b*) those of the side chains. For the high-resolution structures in the R1 bin, the main features of the distributions may be summarized as a tight distribution of the buried amino acids, wider and mutually similar distributions of amino acids in contact with another protein or with DNA, large fluctuations of the phosphodiester backbone of uncomplexed DNA residues and a large difference between water molecules bridging protein and DNA residues and waters on the protein surface. Comparison of Fig. 2 ▶ and Supplementary Fig. S2(*c*) then confirms that both scaling formulas show a similar smearing of the differences observed at the highest resolution.

### 3.6. *B* factors for different groups of structures   

In our previous work (Schneider *et al.*, 2014 ▶), we sorted protein–DNA complexes into various functional groups, and some of the groups contained enough structures in the three resolution bins to be analyzed separately. Of these, the *B*-factor distributions for DNA complexes of transcription factors and nucleases are shown in Supplementary Figs. S1(*c*)–S1(*f*). Analysis of the distributions revealed that all significant features of the *B*-factor distributions discussed above for all structures were also valid for these specific types of complexes. The similarities included the differences between different groups of residues, the unique features of buried amino-acid residues, the low *B*-factor values for water bridges and the removal of differences between different residues for lower resolution structures, and also the cooling of amino-acid and nucleotide residues upon their interaction.

## 4. Conclusions   

An analysis of scaled *B*-factor distributions in over 700 crystal structures of protein–DNA complexes showed that the dynamics of biopolymer residues, amino acids and nucleotides, as well as ordered water molecules, is first of all a function of their neighbourhood: amino acids in the interior of proteins have the tightest distribution of their displacements, residues forming the biopolymer interfaces have an intermediate distribution and residues exposed to the solvent have the widest distribution (Fig. 1 ▶). This general picture is valid in all of the three resolution bins studied here, but the differences are most pronounced in the highest resolution structures (Fig. 2 ▶ and Supplementary Figs. S1 and S2).

Of all residue types, the lowest *B* factors and a relatively low variance of their distributions was observed for buried amino acids (buried aa), and these residues have another property that distinguishes them from all others: their backbone and side chains show virtually identical distributions. The other extreme is formed by the DNA backbone: it has a high flexibility even when complexed with proteins (DNA–protein nt), but the distributions of uncomplexed DNA backbone (exposed nt) are extremely wide and are comparable to those of surface water even in the group of high-resolution structures (≤1.9 Å; bin R1).

Distributions were calculated by two scaling methods, unity-based (1) and *z*-score (2), and both show the same general trends. The distributions showed that the high-resolution data reflect the expected properties of biomolecular residues but that the *B*-factor distributions of lower resolution structures become wider and have higher median values when the crystallographic resolution becomes lower. There is one important exception to this trend: both types of analyzed water residues, bridge w and surface w. In the lowest resolution bin R3, bridge waters become almost as stiff as buried amino acids, and interface waters are stiffer than amino acids at the protein–protein structure. In our opinion, these observations can be viewed as a refinement artifact rather than a reflection of the physical reality.

Higher *B*-factor values and widening of the distributions of solvent-exposed residues relative to their interacting counterparts is significant at 50–75%, and while this percentage slightly diminishes at low resolution the difference remains highly significant in all cases. The entropic cost of complexation must therefore be considerable and becomes more obvious when one considers the low *B* factors of the bridge waters.

The present overview of the behaviour of *B* factors demonstrates that the *B* factors of high-resolution structures reflect the expected dynamics of residues in protein–DNA complexes but that the *B* factors of lower resolution structures should be treated cautiously.

## Supplementary Material

Supplementary Figure S1.. DOI: 10.1107/S1399004714014631/dz5328sup1.pdf

Supplementary Figure S2.. DOI: 10.1107/S1399004714014631/dz5328sup2.pdf

Supplementary Table 1.. DOI: 10.1107/S1399004714014631/dz5328sup3.xlsx

## Figures and Tables

**Table 1:** Medians of the *B* factors scaled by the unity-based algorithm (1) and the numbers of amino acids, nucleotides and water residues in various environments The residues were extracted from the structures of nonredundant protein–DNA complexes with data-collection temperature lower than 180 K sorted into three resolution bins. Bin R1 contains 165 structures with a crystallographic resolution of 1.9 Å or better, bin R2 contains 357 structures with resolution 1.9–2.5 Å and bin R3 contains 187 structures with resolution 2.5–3.0 Å. The median values listed in this table were calculated for the backbone (BB) atoms of the residues. More complete statistics can be found in Supplementary Table S1.

	Resolution ≤1.9 Å		Resolution 1.9–2.5 Å		Resolution 2.5–3.0 Å	
Type of residue†	Median‡	Residues§	Median‡	Residues§	Median‡	Residues§
buried aa	14	19085	20	46934	26	33693
exposed aa	32	6531	39	18687	45	15025
partially buried aa	—	26107	—	70042	—	53510
protein–protein aa	21	2202	23	7258	29	5240
protein–symprotein aa	—	2693	—	5799	—	3744
protein–DNA aa	19	2625	23	8147	29	5685
DNA–protein nt	31	1737	35	5726	36	4146
exposed nt	51	2120	54	7676	54	5240
bridge w	28	3370	27	6285	27	1109
surface w	52	10878	44	16360	33	3267

† Definitions of the residue classes are given in §[Sec sec2]2.

‡ Median values are listed for the backbone atoms; the medians for the side-chain atoms can be found in Supplementary Table S1.

§ Numbers of residues in the listed classes.

## Footnotes

1 Supporting information has been deposited in the IUCr electronic archive (Reference: DZ5328).
