# Supplementary material for: Local dynamics of proteins and DNA evaluated from crystallographic B factors
Source: Acta Crystallogr D Biol Crystallogr. 2014 Aug 29;70(Pt 9):2413–9. doi: 10.1107/S1399004714014631 (PMC4157449; doi:10.1107/S1399004714014631)
Supplement: Supplementary file 1 [file d-70-02413-sup1.pdf]

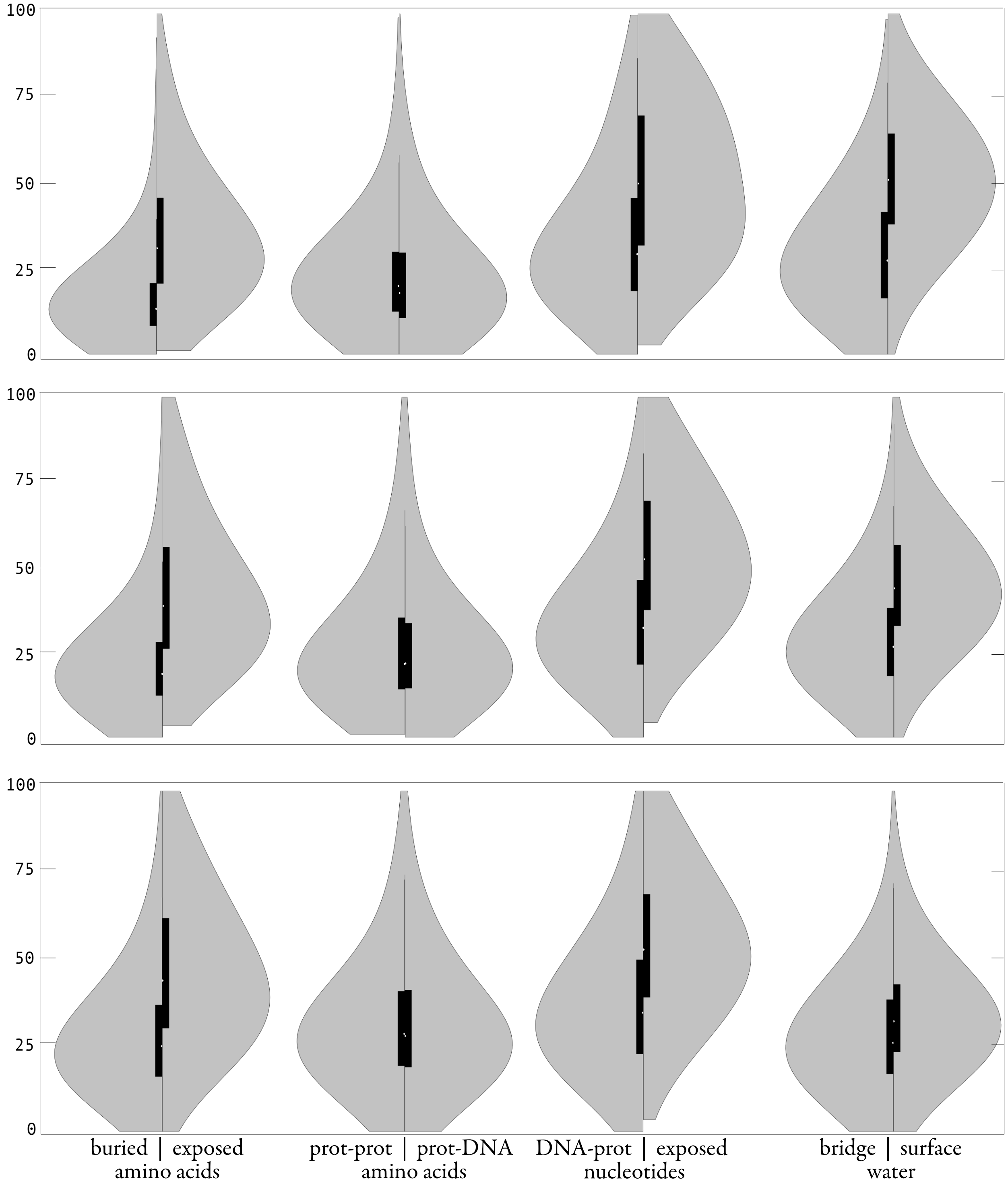

Figure S1 a) B-factor distributions of the backbone atoms. B-factors were scaled between 1 and 100 by unity-based normalization.
Top row: 165 structures with resolution better than 1.9 Å (bin R1).
Middle row: 357 structures with resolution 1.9-2.5 Å (bin R2).
Bottom row: 187 structures with resolution 2.5-3.0 Å (bin R3).
Water bridges considered between amino acids and DNA phosphates.

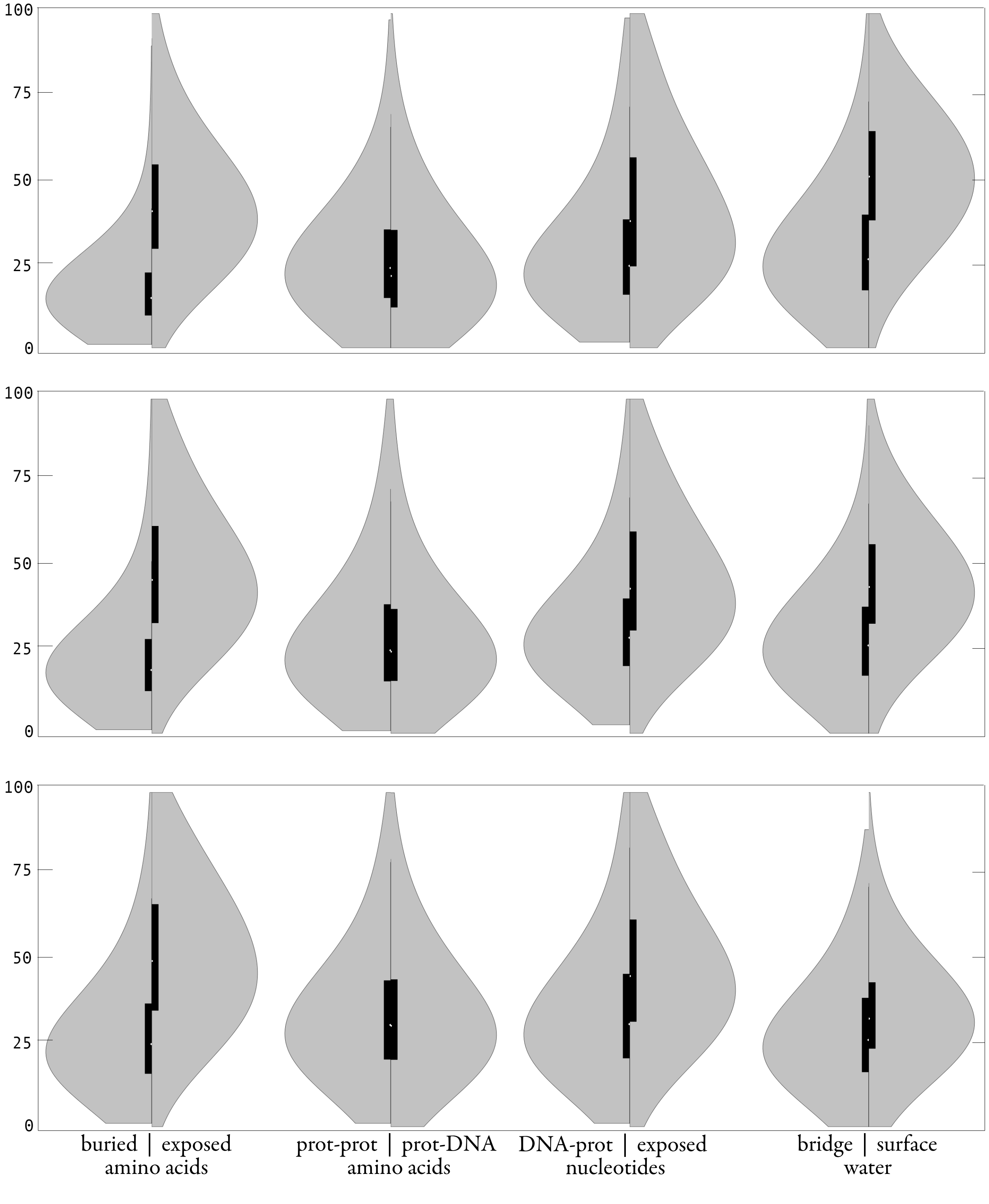

Figure S1 b) B-factor distributions of the side chain atoms. B-factors were scaled between 1 and 100 by unity-based normalization.
Top row: 165 structures with resolution better than 1.9 Å (bin R1).
Middle row: 357 structures with resolution 1.9-2.5 Å (bin R2).
Bottom row187 structures with resolution 2.5-3.0 Å (bin R3).
Water bridges considered between amino acids and DNA bases.

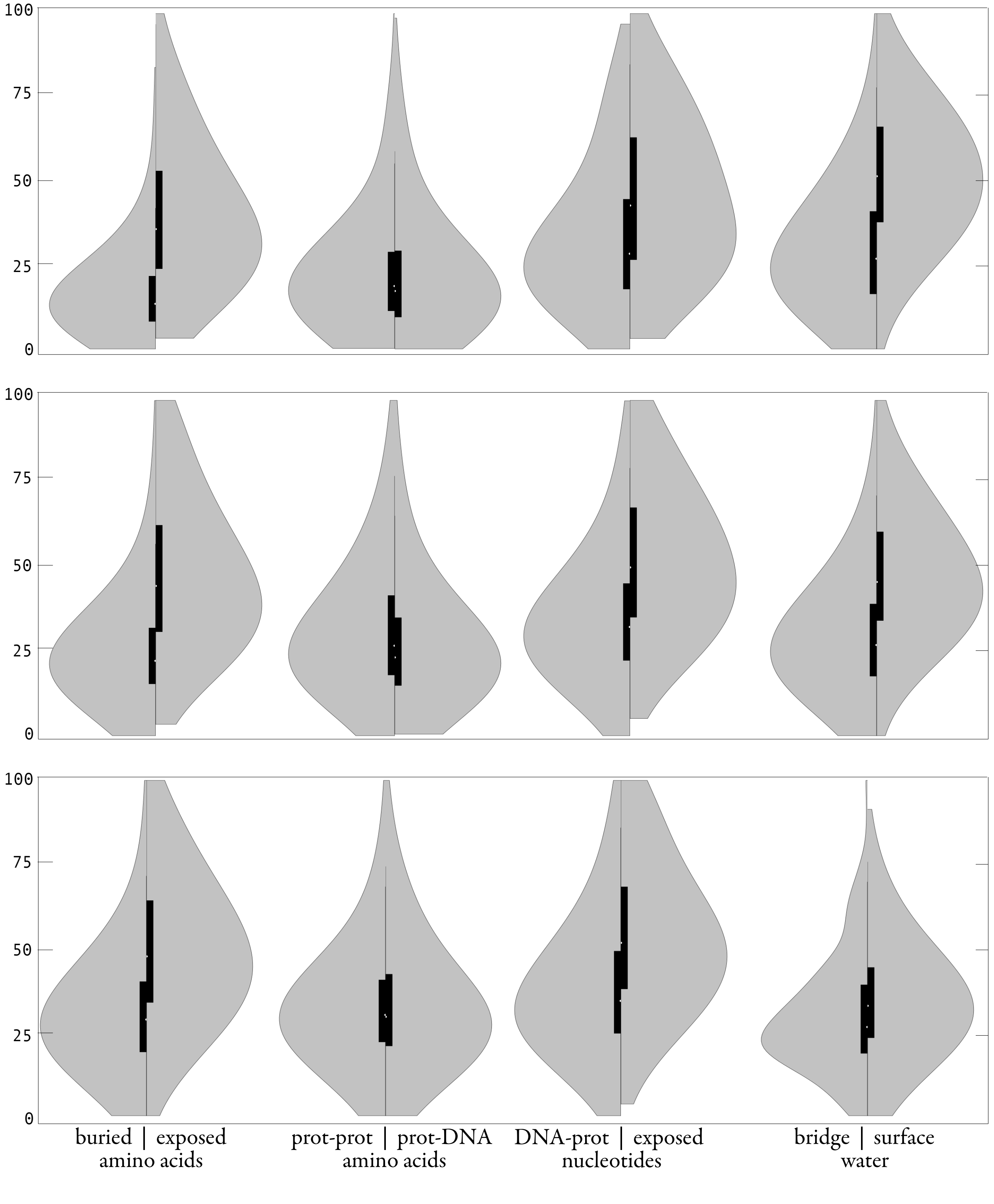

Figure S1 c) B-factor distributions of the backbone atoms of the protein-DNA complexes containing transcription factors.
B-factors were scaled between 1 and 100 by unity-based normalization.
Top row: 56 structures with resolution better than 1.9 Å (bin R1).
Middle row: 132 structures with resolution 1.9-2.5 Å (bin R2).
Bottom row: 89 structures with resolution 2.5-3.0 Å (bin R3).
Water bridges considered between amino acids and all DNA atoms.

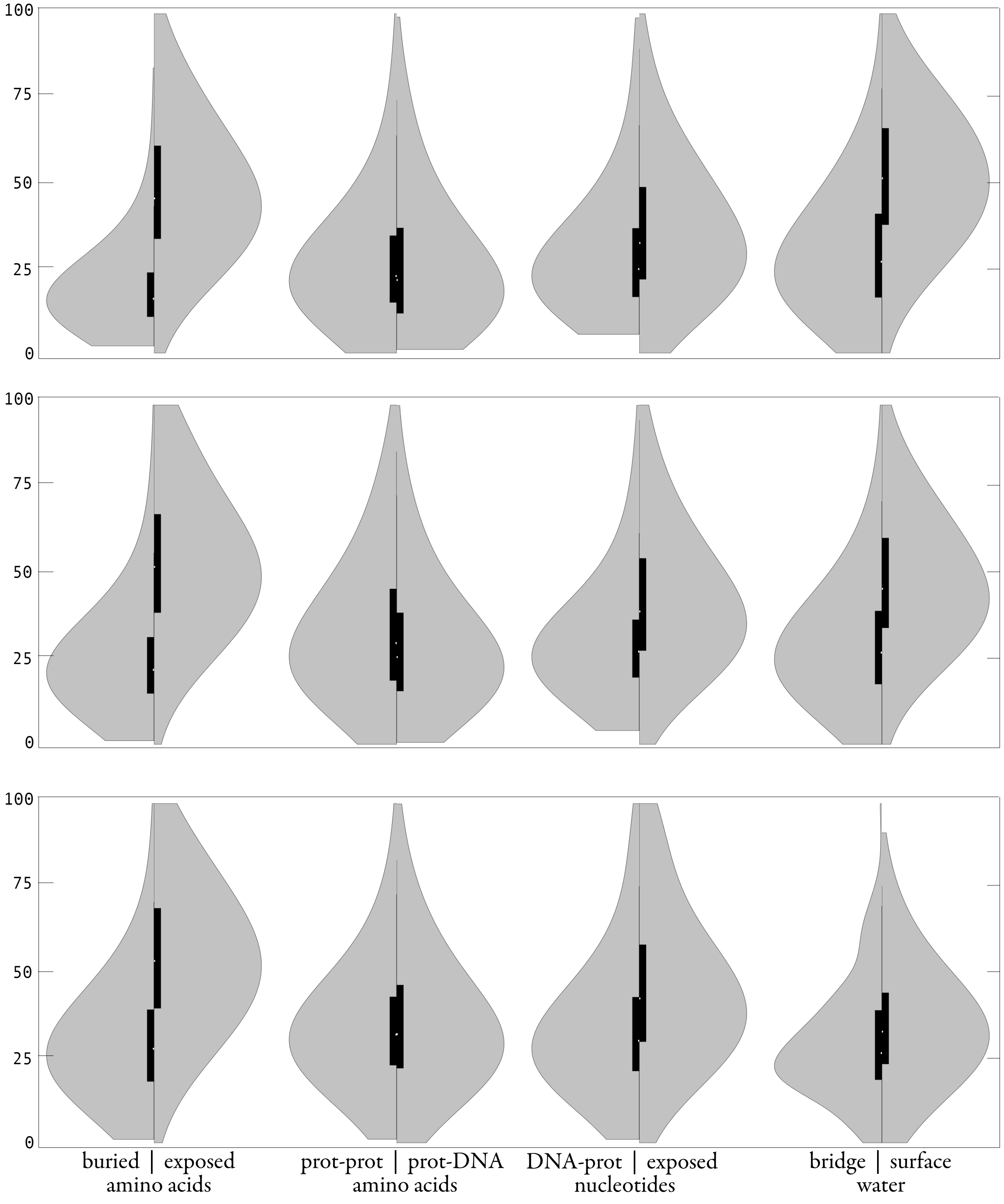

Figure S1 d) B-factor distributions of the side chain atoms of the protein-DNA complexes containing transcription factors.
B-factors were scaled between 1 and 100 by unity-based normalization.
Top row: 56 structures with resolution better than 1.9 Å (bin R1).
Middle row: 132 structures with resolution 1.9-2.5 Å (bin R2).
Bottom row: 82 structures with resolution 2.5-3.0 Å (bin R3).
Water bridges considered between amino acids and all DNA atoms.

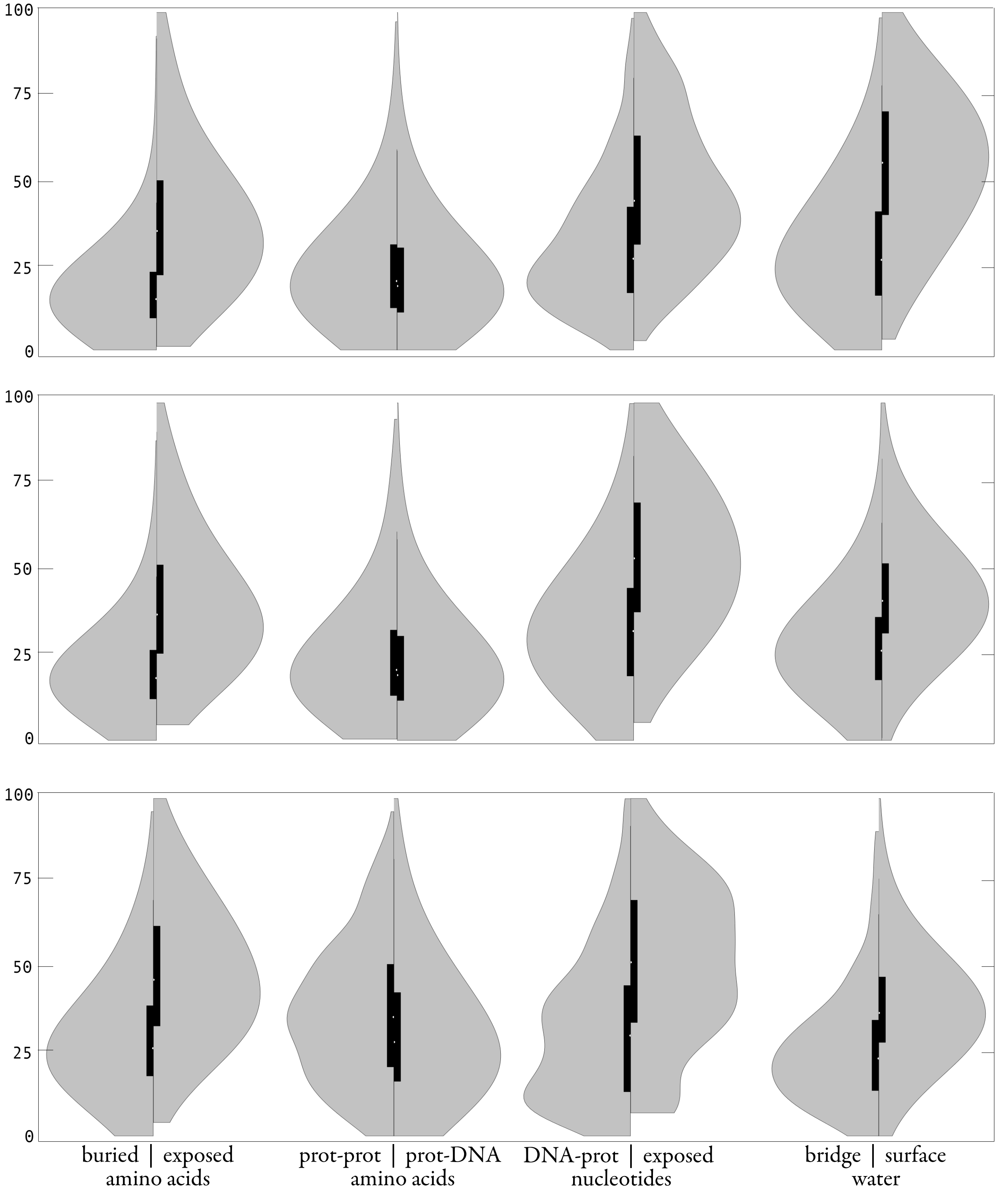

Figure S1 e) B-factor distributions of the backbone atoms of the protein-DNA complexes containing nucleases.
B-factors were scaled between 1 and 100 by unity-based normalization.
Top row: 41 structures with resolution better than 1.9 Å (bin R1).
Middle row: 59 structures with resolution 1.9-2.5 Å (bin R2).
Bottom row: 21 structures with resolution 2.5-3.0 Å (bin R3).
Water bridges considered between amino acids and all DNA atoms.

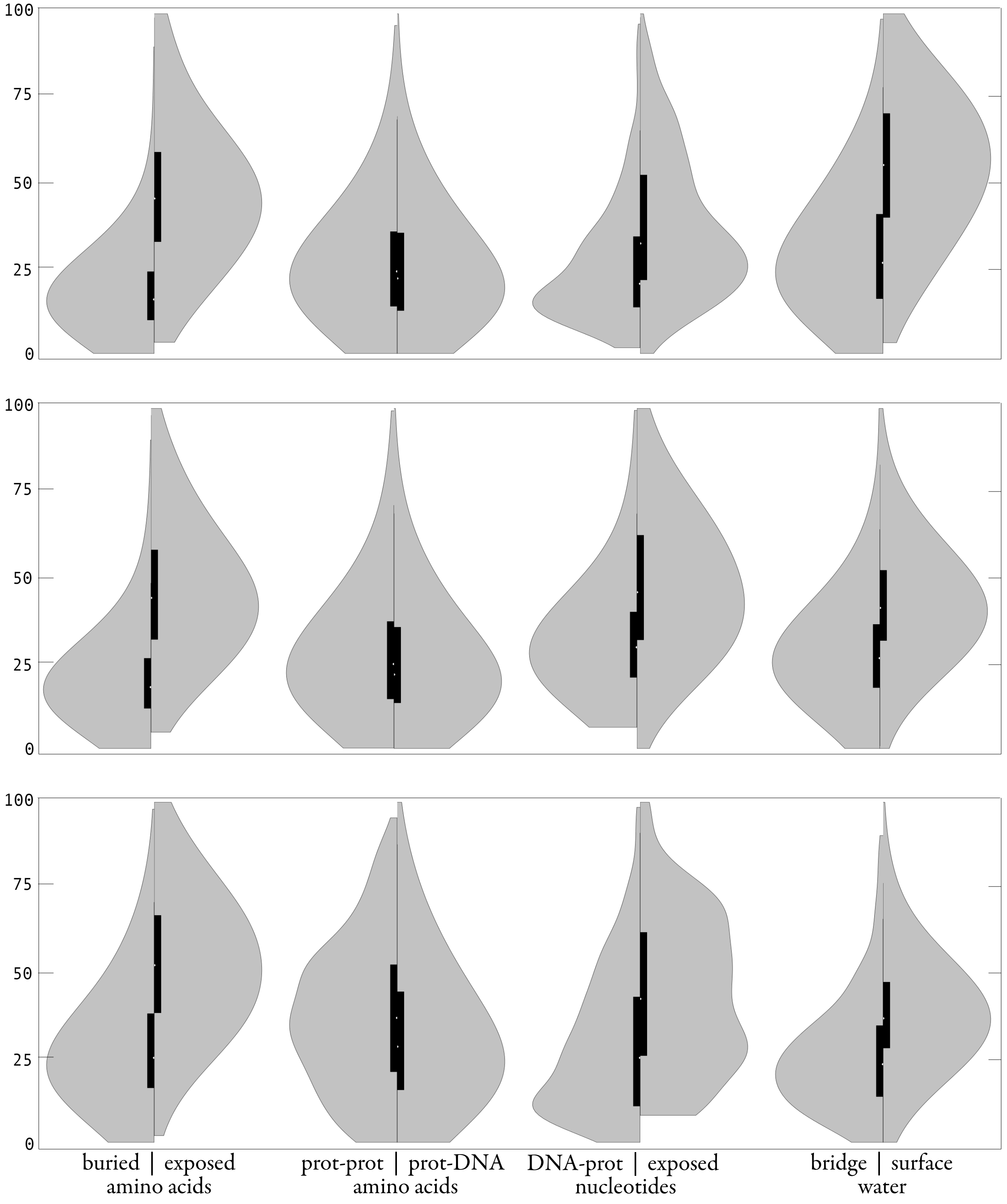

Figure S1 f) B-factor distributions of the side chain atoms of the protein-DNA complexes containing nucleases.
B-factors were scaled between 1 and 100 by unity-based normalization.
Top row: 41 structures with resolution better than 1.9 Å (bin R1).
Middle row: 59 structures with resolution 1.9-2.5 Å (bin R2).
Bottom row: 21 structures with resolution 2.5-3.0 Å (bin R3).
Water bridges considered between amino acids and all DNA atoms.
