# Supplementary material for: Local dynamics of proteins and DNA evaluated from crystallographic B factors
Source: Acta Crystallogr D Biol Crystallogr. 2014 Aug 29;70(Pt 9):2413–9. doi: 10.1107/S1399004714014631 (PMC4157449; doi:10.1107/S1399004714014631)
Supplement: Supplementary file 2 [file d-70-02413-sup2.pdf]

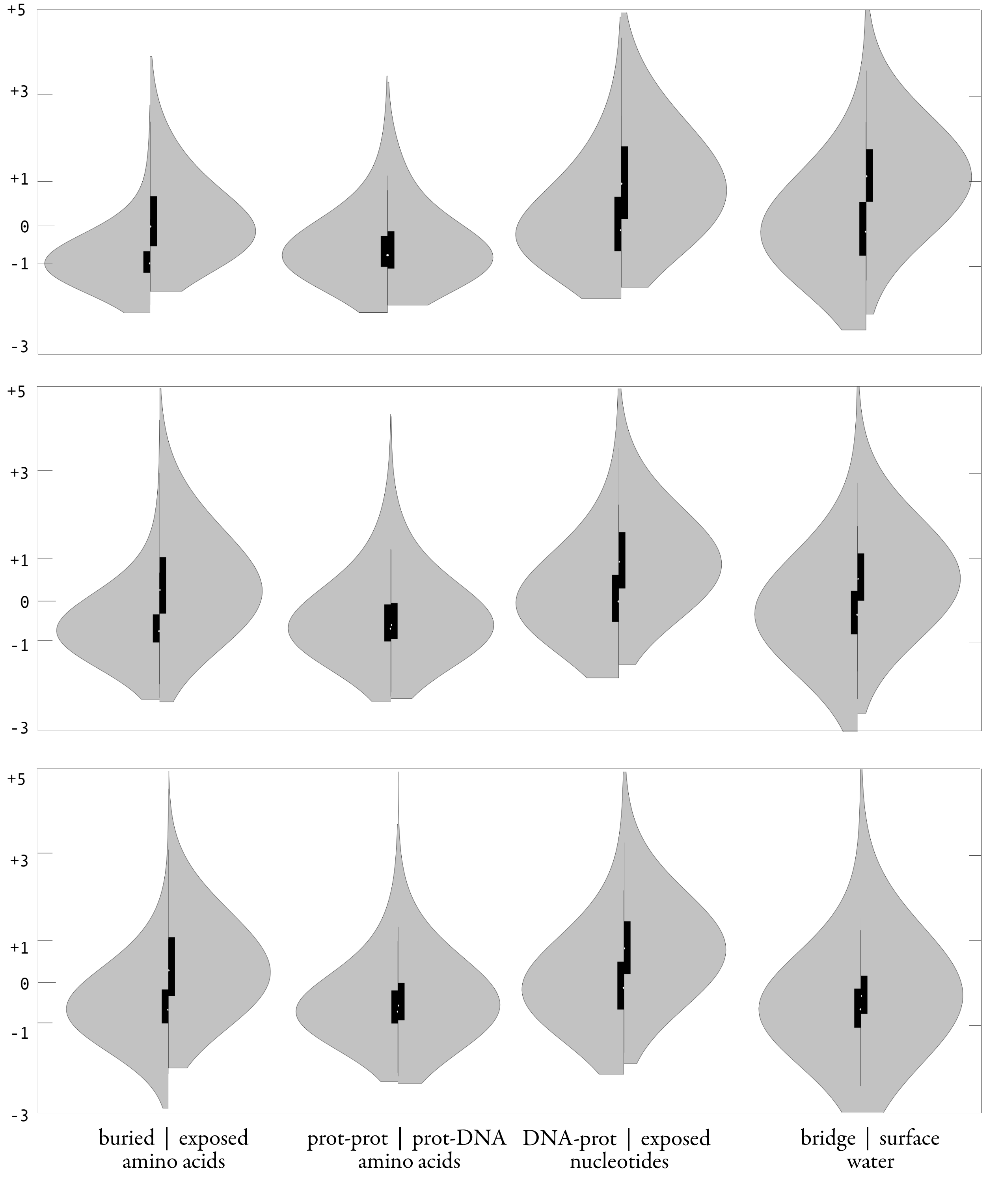

Figure S2 a) B-factor distributions of the backbone atoms of the B-factors normalized as z-scores.
Top row: 165 structures with resolution better than 1.9 Å (bin R1).
Middle row: 357 structures with resolution 1.9-2.5 Å (bin R2).
Bottom row: 187 structures with resolution 2.5-3.0 Å (bin R3).
Water bridges considered between amino acids and DNA phosphates.

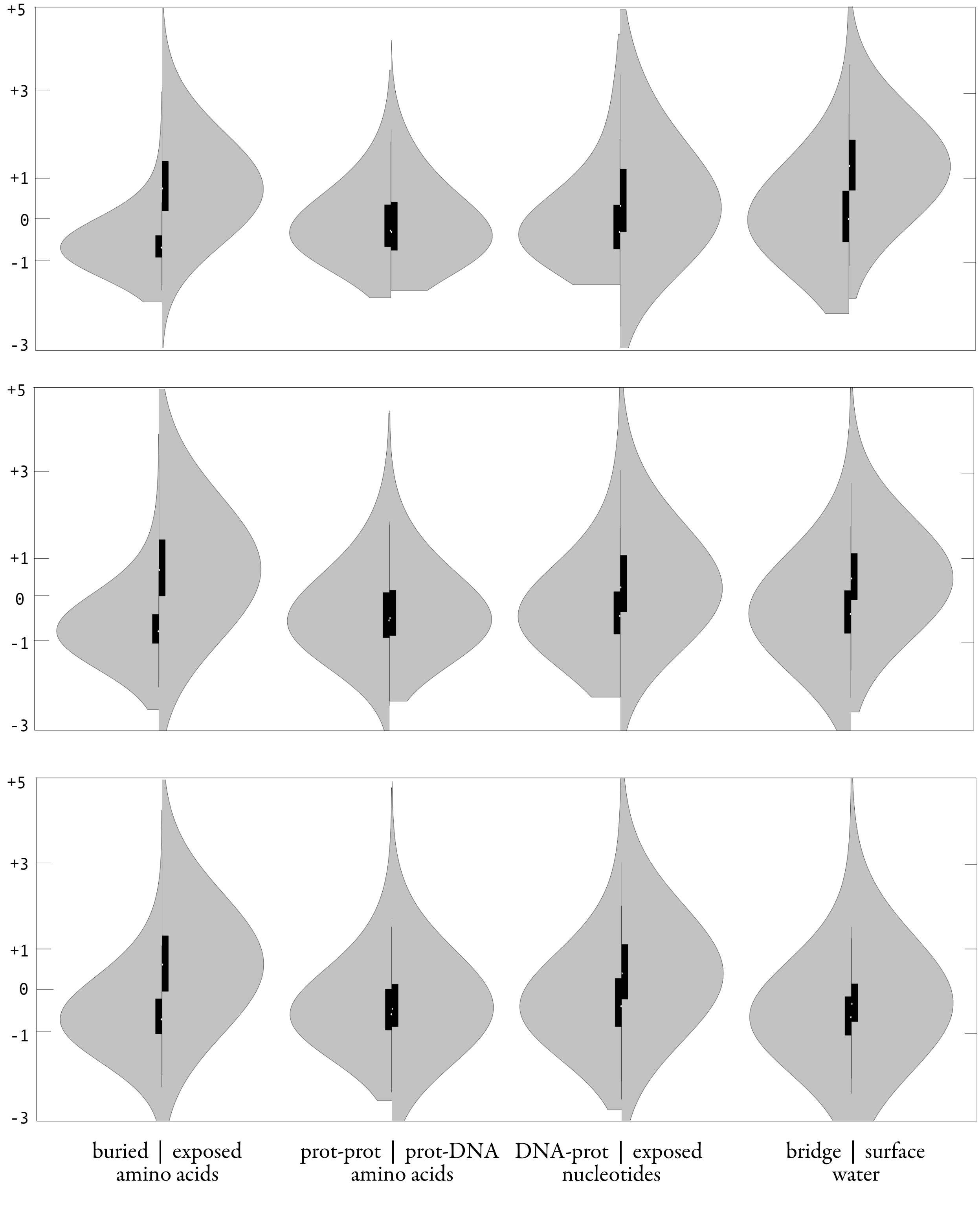

Figure S2 b) B-factor distributions of the side chain atoms of the B-factors normalized as z-scores.
Top row: 165 structures with resolution better than 1.9 Å (bin R1).
Middle row: 357 structures with resolution 1.9-2.5 Å (bin R2).
Bottom row:187 structures with resolution 2.5-3.0 Å (bin R3).
Water bridges considered between amino acids and DNA bases.

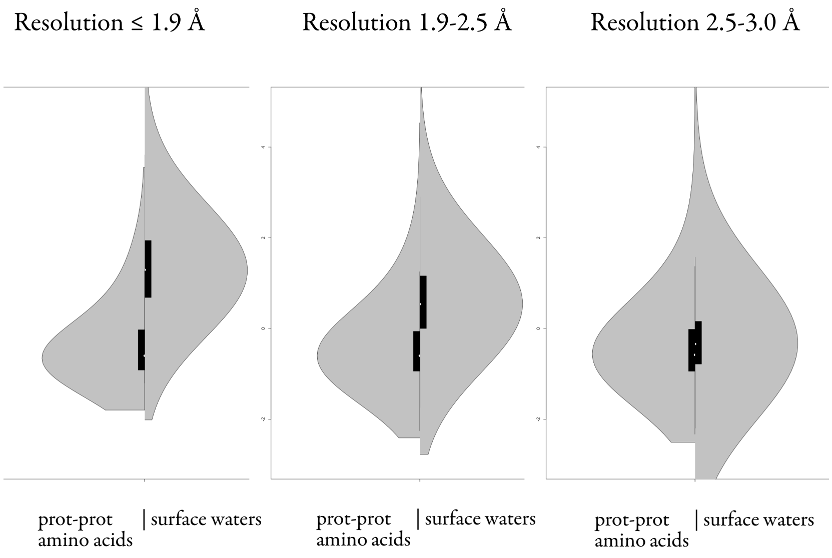

Figure S2 c) Comparison of normalized B-factor distributions (normalized as z-scores) in the three resolution bins for the backbone atoms of amino acids in contact with another protein (group of residues *protein-protein aa*) and the first-shell water molecules (group *surface w*).
